# Progressive Sensorineural Hearing Loss Following Cisplatin Chemotherapy: Mechanisms Underlying Cochlear Retention and Long-Term Ototoxicity

**DOI:** 10.3390/ph19050779

**Published:** 2026-05-15

**Authors:** Antonio Ruggiero, Pasqualina Maria Picciotti, Stefano Mastrangelo, Alberto Romano, Dario Talloa, Jacopo Galli, Giorgio Attinà

**Affiliations:** 1 Pediatric Oncology Unit, Fondazione Policlinico Universitario Agostino Gemelli IRCCS, 00168 Rome, Italy; stefano.mastrangelo@unicatt.it (S.M.); alberto.romano@policlinicogemelli.it (A.R.); dario.talloa@guest.policlinicogemelli.it (D.T.); giorgio.attina@policlinicogemelli.it (G.A.); 2 Department of Woman and Child Health, Università Cattolica del Sacro Cuore, 00168 Rome, Italy; 3 Unit of Otorhinolaryngology—Head and Neck Surgery, Fondazione Policlinico Universitario Agostino Gemelli IRCCS, 00168 Rome, Italy; pasqualinamaria.picciotti@policlinicogemelli.it (P.M.P.); jacopo.galli@unicatt.it (J.G.); 4 Department of Head and Neck and Sensory Organs, Università Cattolica del Sacro Cuore, 00168 Rome, Italy

**Keywords:** cisplatin, ototoxicity, sensorineural hearing loss, cochlea, stria vascularis, oxidative stress, cochlear pharmacokinetics, synaptopathy, platinum retention

## Abstract

Cisplatin-induced ototoxicity is a permanent, bilateral sensorineural hearing loss occurring in up to 80% of treated patients. Its defining and clinically challenging feature is the progressive worsening of auditory function that continues well after chemotherapy has ended, a trajectory that cannot be explained by cumulative dose alone. This article is a comprehensive review of the present research studies on mechanisms that are responsible for this post-treatment progression. The cochlea, unlike other organs, appears to be unable to eliminate platinum (the active divalent metal ion released from cisplatin and responsible for its cytotoxic and ototoxic effects): traces of it can be found in human temporal bone tissue even more than 18 months after last infusion, and bone might serve as a long-term systemic reservoir. Within the inner ear, platinum accumulates preferentially in the stria vascularis, impairing endocochlear potential and outer hair cell function. Retained platinum sustains cascading effects including sustained NOX3-dependent oxidative stress, mitochondrial dysfunction, ongoing genotoxic injury to non-regenerative cells, and the early loss of ribbon synapses that precipitates delayed spiral ganglion neurodegeneration. Pharmacogenetic variability in platinum transport and antioxidant metabolism further modulates individual susceptibility. These findings support lifelong audiological surveillance and provide a basis for designing strategies that can protect hearing without compromising the essential anticancer efficacy of cisplatin therapy.

## 1. Introduction

Since its approval by the FDA in 1978, cisplatin (cis-diamminedichloroplatinum II) has been a cornerstone of oncological chemotherapy and continues to play a significant role in treatment regimens of malignancies of the head and neck, lung, bladder, ovary, and testis, and in a wide range of pediatric malignancies [1,2]. The antineoplastic effect of the drug is mainly due to the formation of both intrastrand and interstrand DNA cross-links that inhibit DNA replication and transcription, which eventually leads to the occurrence of apoptosis in tumor cells that divide rapidly [3]. However, cisplatin has dose-limiting toxicities on various organ systems, with the most common being the kidney, the peripheral nervous system, and, most intractably, the inner ear. Cisplatin-induced ototoxicity (CIO) is a bilateral, symmetrical, progressive, and permanent sensorineural hearing loss (SNHL) that starts at high frequencies and progresses down to lower frequencies with increasing cumulative dose [4,5]. The reported incidence rates differ significantly across studies (between about 20–80 percent in adults and between 22–70 percent in pediatric populations), depending on the cumulative dose given, patient age, and the audiological criterion used to determine clinically significant hearing impairment [6,7,8,9]. Tinnitus is also common, and this further undermines the quality of life of the patients [10]. A particularly clinically challenging feature of CIO is its propensity to worsen after the completion of treatment. Several clinical studies, predominantly in pediatric populations, have shown that hearing loss measured by pure-tone audiometry continues to get worse for months or even years after the last dose of cisplatin has been given [11,12]. This delay and steady worsening of the condition cannot be uniformly accounted for by a direct dose–response relationship alone and therefore, researchers are investigating additional pathophysiological mechanisms. Understanding these mechanisms will have an immediate lead on practical aspects: it will guide the creation of hearing monitoring programs, allow for the identification of patients most at risk, and may even suggest new therapeutic targets to prevent or reduce cisplatin-induced hearing loss. Regarding the pharmacokinetics of cisplatin, the drug is administered intravenously and distributed rapidly throughout the body. It undergoes aquation in low-chloride environments, releasing a reactive platinum species that binds preferentially to plasma proteins; only the free, unbound fraction is pharmacologically active. Cisplatin is primarily eliminated by the kidney, with a biphasic plasma half-life consisting of an initial distribution phase (t½α ≈ 20–30 min) and a prolonged terminal elimination phase (t½β ranging from several days to weeks for protein-bound platinum), which underpins its propensity for long-term tissue retention [3]. The primary target organ for ototoxicity is the cochlea, the snail-shaped, fluid-filled bony structure of the inner ear responsible for converting sound vibrations into neural signals. Within the cochlea, three main functional compartments are relevant to cisplatin toxicity: the stria vascularis (a highly vascularized epithelium lining the lateral wall of the scala media that generates and maintains the endocochlear potential essential for outer hair cell mechanotransduction), the organ of Corti (where outer hair cells and inner hair cells are arranged tonotopically along the basilar membrane), and the spiral ganglion (which contains the primary afferent auditory neurons that relay signals to the brainstem). Cisplatin preferentially damages the basal turn of the cochlea, the region encoding high-frequency sounds, explaining the characteristic high-frequency onset of ototoxicity [4,5]. This review aims to integrate and analyze existing knowledge about the mechanisms underlying the progressive nature of cisplatin ototoxicity. We examined the cochlear pharmacokinetics and the permanent retention of platinum in the inner ear, the selective build-up of cisplatin in the stria vascularis and its effects on function. In addition, we reviewed the molecular pathways of oxidative stress and inflammation, with new evidence suggesting that cochlear synaptopathy is a cause of progressive neuropathy, the impact of genetic susceptibility, and the implications of these findings for medical practice and research.

This narrative review was performed using a systematic search of Pub-Med/MEDLINE and Embase from inception to March 2026, with the following terms: “cisplatin”, “ototoxicity”, “sensorineural hearing loss”, “cochlea”, “platinum retention”, “stria vascularis”, “oxidative stress”, “cochlear synaptopathy”, “pharmacokinetics”, and “otoprotection”, used either alone or in combinations. No restrictions were placed on language or publication date. This search retrieved original articles, reviews and systematic reviews, meta-analyses, and clinical trials. The search results were filtered for relevance based on title and abstract. The abstracted articles were then reviewed for relevance and inclusion. The searches retrieved articles describing mechanisms of hearing loss following cisplatin, cochlear pharmacokinetics, molecular pathways involved in a-hearing loss, genetic susceptibilities, and otoprotective measures. Reference lists of included articles were manually screened to identify additional relevant publications.

## 2. Epidemiology and Clinical Characteristics of Cisplatin Ototoxicity

### 2.1. Incidence and Risk Factors

Estimates of CIO incidence are highly variable, primarily because of differences in the audiological tests employed, the grading scales used (Brock criteria, SIOP Boston scale, Chang scale, Common Terminology Criteria for Adverse Events), the frequencies evaluated, and the patient populations studied [13,14]. In a large pediatric cohort study (n = 102), Yancey et al. (2012, USA; multi-center pediatric study) reported an overall incidence of cisplatin-related ototoxicity of 42%, with 28% of patients showing moderate to severe impairment (Brock score ≥ 2) [13]. Bertolini et al. (2004, France; single-center pediatric cohort), in a series of 120 children with a median follow-up of seven years, documented grade 2 or above hearing loss in 37% of those treated with cisplatin alone [12]. Tang et al. described an incidence range of 20–70% in children across published studies, highlighting the universal association with oxidative stress mechanisms and activation of the apoptotic pathway in the cochlear tissue [5]. In a landmark series of adult cancer survivors, Frisina and colleagues tested hearing in 488 men who had germ cell tumors. Eighty percent showed hearing loss as defined by the American Speech-Language-Hearing Association (ASHA), and 18% had severe to profound loss. Tinnitus was reported in 40% of the patients. For every 100 mg/m^2^ increase of cisplatin given, hearing thresholds worsened by 3.2 dB at 4 kHz and above (*p* < 0.001) [6].

The major risk factors associated with the development of CIO include the overall total cumulative dose of cisplatin administered to the patient (the most reliable predictor of CIO across all age groups); younger age at time of treatment (children, especially those less than 5 years old, are at significantly greater risk of CIO compared to adults) [7,8,15,16,17]; concurrent or prior cranial radiotherapy, which potentiates cochlear damage through additive free-radical generation; renal dysfunction prior to receiving cisplatin could lead to prolonged duration and/or elevated levels of systemic exposure to cisplatin; simultaneous administration of other nephrotoxic/ototoxic drugs such as aminoglycoside antibiotics or loop diuretics; and genetic predisposition to develop cytotoxicity from chemotherapeutic agents (Table 1) [6,7,8,9,16,17,18,19].

### 2.2. Audiological Characteristics

The audiological signature of CIO is a high-frequency, bilateral, predominantly symmetric sensorineural hearing loss that typically begins at 8 kHz and extends progressively toward lower frequencies as the cumulative drug exposure increases [4,36]. This tonotopic pattern of cochlear injury reflects the preferential susceptibility of the outer hair cells in the basal turn of the cochlea, which respond to high-frequency sounds, and parallels the distribution of cisplatin accumulation within cochlear tissue as demonstrated by pharmacokinetic studies [37,38]. In severely affected patients, hearing loss can extend to frequencies below 8 KHz, which are critical for speech discrimination, with potential impairment of social hearing [39]. Bertolini et al. (n = 120 children, median follow-up 7 years) documented a striking post-treatment progression of cochlear damage: whereas only 5% of audiograms showed grade ≥ 2 toxicity (Brock’s grading system) at the end of therapy, this proportion rose to 11% in early post-treatment evaluations and reached 44% after more than two years of follow-up; grade 3–4 ototoxicity was ultimately observed in 15% of patients [12]. Fetoni et al., in a cohort of 160 children treated with platinum compounds over seven years, confirmed an overall ototoxicity incidence of 25% (SIOP Boston scale) and reported that 8.6% of patients showed further audiometric deterioration after the end of chemotherapy, underscoring that post-treatment surveillance is mandatory [15]. Similarly, Kolinsky et al. documented late-onset hearing loss as a significant complication in long-term cancer survivors, with progressive deterioration sometimes first becoming clinically apparent years after treatment cessation [11]. Extended high-frequency audiometry, monitoring thresholds at 10–16 kHz, has been shown to detect ototoxic changes earlier than standard audiometry and should be incorporated into monitoring protocols [40].

## 3. Pharmacokinetics and Tissue Distribution of Cisplatin

### 3.1. Organ Distribution and Elimination Kinetics

The pharmacokinetic behaviour of cisplatin in the cochlea is fundamentally different from that in other body organs and represents a central mechanism driving the progressive nature of its ototoxicity. In the study by Breglio et al., inductively coupled plasma mass spectrometry (ICP-MS) was employed to quantify platinum distribution in multiple organs of mice following a clinically analogous-multisession cisplatin regimen [37]. In most organs including the kidney, liver, lung, and heart, platinum levels peaked shortly after each injection cycle and then declined substantially during the recovery periods, following expected pharmacokinetic elimination curves. In contrast, the cochlea exhibited a strikingly different pattern: platinum accumulated progressively with each successive treatment cycle, with no measurable elimination during the intervening recovery intervals, indicating an extremely limited capacity for drug clearance from this compartment. Moreover, when platinum levels in various organs were compared between the end of the full treatment regimen (day 42) and a subsequent 60-day recovery period, significant elimination of platinum was observed in all organs except the cochlea and the femur. Platinum levels in the cochlea remained essentially unchanged over the 60-day post-treatment period, demonstrating indefinite retention of the drug within the inner ear [37]. The cochlea’s limited ability to eliminate cisplatin is likely attributable to the unique properties of the blood–labyrinth barrier (BLB), which—unlike the blood–brain barrier— appears to permit cisplatin entry while potentially limiting its efflux [28].

### 3.2. Retention in Human Cochlear Tissue

The translational relevance of these pharmacokinetic findings was confirmed by the analysis of post-mortem human temporal bone specimens from patients who had received cisplatin chemotherapy. ICP-MS measurements of cochlear sections from cisplatin-treated patients consistently revealed significantly higher platinum concentrations compared with sections from age- and sex-matched controls who had not received the drug [37]. Importantly, platinum was detectable in cochlear tissue up to at least 18 months after the patient’s last cisplatin infusion. The only pediatric patient included in the present analysis had cochlear platinum concentrations that were greater than all the adult patients, even though they had received a lower cumulative dose, which might suggest that pharmacokinetic differences in cochlear platinum based on age may be partly responsible for the increased risk of developing auditory impairments as a direct result of receiving chemotherapy [37]. In addition, the results are consistent with previous studies by Tothill et al. [41] and Sprauten et al. [35] that reported elevated platinum concentrations in human tissue as much as several years after the termination of cisplatin therapy, and retrospective data from testicular cancer survivors that demonstrate long-term increases in circulating platinum concentrations for decades after treatment [42,43]. Therefore, the persistence of platinum in the cochlea can contribute to prolonged loss of hearing after the cessation of treatment because there continues to be an exposure to cochlear tissue of platinum following systemic elimination of the compound.

### 3.3. Platinum Retention in Bone: A Systemic Reservoir

Outside the cochlea, the long bones also exhibited significant platinum retention in the mouse model, with femoral levels comparable to cochlear levels at 60 days post-treatment [37]. This finding is consistent with the demonstrated capacity of cisplatin to bind extensively to type I collagen, the principal structural protein of bone, from which it dissociates slowly over time [37,44]. Bone may therefore function as a systemic reservoir for platinum, releasing the drug back into the circulation over extended periods [45]. Slow re-mobilization of platinum from bone into the circulation could provide continued low-level cochlear exposure long after the completion of treatment, potentially contributing to the delayed progression of hearing loss observed clinically.

## 4. Accumulation in the Stria Vascularis and Endocochlear Potential Disruption

### 4.1. The Stria Vascularis as the Primary Site of Cisplatin Accumulation

**Figure 1:**
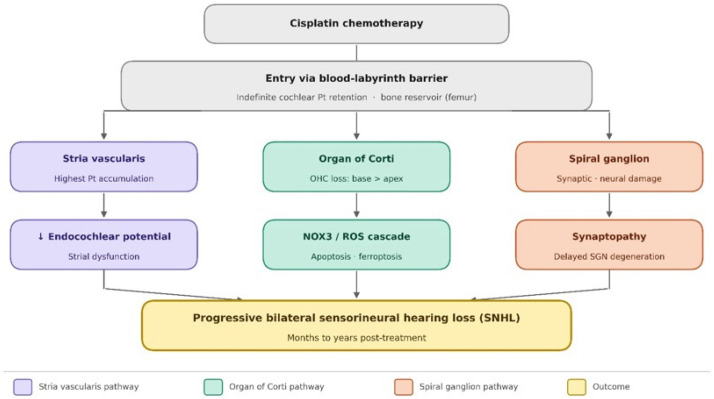
Mechanisms of progressive cisplatin-induced hearing loss. Pathways underlying progressive cisplatin-induced hearing loss are as follows. Cisplatin enters the inner ear via the blood–labyrinth barrier and is retained in cochlear tissue. Within the cochlea, cisplatin accumulates preferentially in the stria vascularis (SV; highest platinum concentration), the organ of Corti (OC), and the spiral ganglion (SGN). Three major mechanistic cascades drive progressive cochlear injury: (1) impairment of the endocochlear potential through strial dysfunction; (2) NADPH oxidase 3 (NOX3)-mediated reactive oxygen species (ROS) production, culminating in outer hair cell (OHC) apoptosis and ferroptosis; and (3) early ribbon synapse loss (synaptopathy) followed by delayed spiral ganglion neuron degeneration. The convergence of these mechanisms results in a progressive bilateral sensorineural hearing loss (SNHL) that may continue for months to years after the cessation of treatment. The femur is shown as a representative site of skeletal platinum retention, reflecting the sampling approach used in murine pharmacokinetic studies (Breglio et al., 2017 [37]), where long bone was used as a proxy for general skeletal deposition. Abbreviations: SV = stria vascularis; OC = organ of Corti; SGN = spiral ganglion neuron; OHC = outer hair cell; IHC = inner hair cell; NOX3 = NADPH oxidase isoform 3; ROS = reactive oxygen species; EP = endocochlear potential; SNHL = sensorineural hearing loss; BLB = blood–labyrinth barrier. The down arrow means disruption.

Within the cochlea, cisplatin does not distribute uniformly. This section examines the selective accumulation of cisplatin within specific cochlear compartments and its functional consequences, extending the pharmacokinetic framework of Section 3 to the level of individual cochlear structures. The analysis of platinum accumulation in microdissected cochlear subregions (the stria vascularis, the organ of Corti, and the spiral ganglion) using ICP-MS has shown that platinum accumulates preferentially in the stria vascularis compared to the other two compartments [37]. These findings have been corroborated and extended by complementary experimental approaches. Hellberg et al. [38], using a comparative pharmacokinetic model, demonstrated that the differential cochlear retention kinetics between cisplatin and oxaliplatin directly explain the greater ototoxicity of cisplatin: unlike oxaliplatin, which is rapidly cleared from cochlear tissue, cisplatin undergoes slow elimination from the stria vascularis, sustaining a prolonged intracochlear platinum burden. In human studies, long-term retention of platinum in tissues has been confirmed at the systemic level: Tothill et al. [41] detected platinum in postmortem tissue samples of cancer patients treated with platinum-based chemotherapy, and Sprauten et al. [35] reported persistently elevated serum platinum concentrations in testicular cancer survivors up to 20 years after treatment. At the clinical level, Laverdière et al. [39] documented audiometric deterioration in pediatric cancer survivors that correlated with cumulative cisplatin dose and persisted years after treatment cessation, providing clinical correlates to the pharmacokinetic evidence of prolonged cochlear platinum retention. Together, these converging lines of evidence from animal models, human postmortem studies, and clinical cohorts establish that the stria vascularis represents the principal site of cochlear platinum accumulation and that its persistence, rather than transient exposure, is the key driver of progressive, post-treatment ototoxicity (Figure 1).

The stria vascularis contained the greatest amount of accumulated platinum by the end of the cisplatin treatment protocol; this correlation remained after the 60-day post-treatment recovery period. However, all three subregions did show a reduction in platinum accumulation during the post-treatment recovery period, with the stria vascularis having the greatest amount accumulated throughout. Spatial mapping of platinum distribution in human cochlear sections using laser ablation ICP-MS confirmed the relatively high-resolution pattern of the accumulation of cisplatin within the different subregions. Across multiple sections from patients treated with cisplatin, the levels of platinum were invariably the most elevated in the stria vascularis and in sections of the modiolus (the central axis of the cochlea), particularly at the interface between the cochlear nerve and the modiolar bone, with lower levels in the organ of Corti [37]. These findings are consistent with those of fluorescent cisplatin analogues in mice, which showed that BODIPY FL-cisplatin accumulated in the stria vascularis while cochlear hair cells had minimal accumulation. This indicates that the stria vascularis serves as more than a site of platinum accumulation and may actively concentrate platinum compounds.

### 4.2. Consequences for Endocochlear Potential and Hair Cell Function

The stria vascularis generates and maintains the endocochlear potential (EP), a positive electrochemical potential (+80 to +100 mV) of the endolymph relative to the perilymph. This electrochemical gradient provides an electrochemical driving force for cochlear hair cells to perform mechanotransduction [46]. An EP disruption will directly impact outer hair cell function since the EP determines the electrochemical driving force for mechanosensory transduction currents through stereociliary bundles. In the mouse model of CIO, recording of the EP during and after cisplatin treatment demonstrated a significant reduction in the endocochlear potential, both at the end of the first treatment cycle and at 60 days following the completion of the full regimen [37]. The repeated reduction in the EP in the stria vascularis due to accumulated platinum damage is a mechanistic rationale for the continuing decline in outer hair cell function (as evidenced by decreased distortion product otoacoustic emission (DPOAE) amplitudes) that continues to decline post cessation of cisplatin therapy. The stria vascularis’s unique vulnerability to cisplatin accumulation mirrors the stria vascularis’s role as the primary vascular entry point in the cochlea; it is the location for solute entry into the endolymph and is therefore subject to prolonged exposure to cisplatin at very high concentrations and does not have a comparable method for detoxifying or exporting cisplatin. Breglio et al. found a gradient of cisplatin signal intensity in the stria vascularis from base to apex, correlating with the established base to apex susceptibility to hair cell injury from cisplatin; this correlation supports the theory of high frequency hearing loss occurring preferentially from higher concentrations of platinum in the stria vascularis at the cochlear base [37].

## 5. Molecular Mechanisms of Cisplatin-Induced Cochlear Cytotoxicity

### 5.1. Reactive Oxygen Species and Oxidative Stress

Once internalized by cochlear cells, cisplatin exerts cytotoxic effects through multiple converging pathways. The most well-studied of these pathways is the generation of reactive oxygen species (ROS) by cisplatin [47,48]. Within the cochlea, cisplatin activates the NADPH oxidase 3 (NOX3) isoform, which is highly expressed in the inner ear and constitutes a major source of superoxide generation following cisplatin exposure [49,50]. Superoxide and its downstream products including hydrogen peroxide, hydroxyl radicals, and peroxynitrite, initiate a cascade of lipid peroxidation, protein nitration, and mitochondrial membrane disruption. The 4-hydroxynonenal (4-HNE) generated from the lipid peroxidation of the polyunsaturated fatty acids (PUFAs) in the cochlea activates the influx of calcium (Ca^2+^) and the subsequent activation of pro-apoptotic pathways [48,51]. Cisplatin also directly affects the function of mitochondria in cochlear cells: it enters the mitochondria, blocks mitochondrial transcription and translation, decreases the mitochondrial membrane potential, and induces the release of cytochrome c, which triggers the intrinsic apoptotic pathway. This mitochondrial dysfunction increases the production of ROS and energy depletion in highly metabolically active cochlear cells, such as outer hair cells and stria vascularis marginal cells. Because of the high metabolic activity of the stria vascularis, the stria vascularis is uniquely vulnerable to oxidative and mitochondrial damage. Animals treated with cisplatin show increased lipid peroxidation in cochlear tissues and a significant reduction in cochlear glutathione (GSH) levels as well as decreased activity of antioxidant enzymes such as catalase, superoxide dismutase, and glutathione peroxidase in the cochlea [47]. Inhibition of NOX3 with transtympanic (i.e., through the ear drum) delivery of siRNA protects against cisplatin-induced hearing loss in animal models, underscoring the importance of the NOX3 pathway in the process of cisplatin-induced ototoxicity [49]. Additionally, recent investigations suggest that ferroptosis, a process of iron-dependent cell death that occurs when cells undergo lipid peroxidation, also mediates cisplatin-induced cochlear cell death, partly through the NRF2 signaling pathway [52].

### 5.2. Inflammation and Immune Cell Recruitment

Inflammation within the cochlea is a significant contributor to the amplification of cisplatin toxicity and plays a role in the progressive nature of CIO. Cisplatin exposure results in increased levels of proinflammatory cytokines and chemokines in the cochlea, along with increasing numbers of macrophages (CD68+) and other immune cells (CD45+) in the stria vascularis, spiral ganglion, and spiral ligament following the administration of cisplatin [53]. Activation of transient receptor potential vanilloid 1 (TRPV1) channels in the cochlear tissue by cisplatin initiates inflammatory signaling pathways. When TRPV1 is pharmacologically inhibited, significant protection against cisplatin damage is observed in animal models [47]. The ongoing presence of platinum within the cochlea, after cessation of treatment with cisplatin, may continue to generate inflammatory signals, which may lead to further tissue damage while contributing to the deterioration of hearing function. An important emerging mechanistic discovery is that tissue-resident macrophages contribute to cisplatin cochleotoxicity. In a multi-session mouse model, Sung et al. showed that PLX3397 (an FDA-approved CSF1R inhibitor) treatment led to substantial protection from hearing loss, improved outer hair cell survival, and decreased platinum deposition in the inner ear [54]. The results indicate that cochlear macrophages actively participate in the uptake of cisplatin into the stria vascularis, and they are a promising therapeutic target for otoprotection. Wang et al. proposed a comprehensive signaling network for how cisplatin enters the inner ear through the blood–labyrinth barrier to disrupt the homeostasis of cochlear endolymph and activate inflammatory signaling within outer hair cells [51]. Endoplasmic reticulum (ER) stress and caspase-12 activation, along with activation of the mitochondrial apoptotic pathway involving caspase-9 and caspase-3, combine to induce programmed cell death in outer hair cells, inner hair cells, and spiral ganglion neurons [51,55]. The ongoing presence of platinum within cochlear tissue after treatment cessation ensures that these processes are not self-limiting with the end of drug administration.

### 5.3. DNA Damage and Apoptosis Inflammation and Immune Cell Recruitment

Through the formation of both inter-strand and intra-strand DNA adducts, cisplatin produces direct genotoxic (sub-lethal) effects by blocking the progression of both DNA and RNA polymerases, challenging DNA damage checkpoint signaling pathways, and ultimately triggering many forms of apoptotic cell death(s) if the damage cannot be repaired [3]. Cochlear cells, particularly outer hair cells which are post-mitotic and lack regenerative capacity in mammals, undergo irreversible loss following significant DNA damage. The absence of meaningful hair cell regeneration within the mammalian cochlear results in permanent structural deficits, leading to irreversible hearing loss as a result of the cell death associated with cisplatin [4,14]. The longitudinal progression of hearing loss can also be attributed to the continued presence of retained platinum within the cochlear tissue, whereby new platinum-DNA adducts can be formed over time and therefore produce continued (but low level) genotoxic stress on all surviving cochlear cells [14,19].

## 6. Cochlear Synaptopathy as a Mechanism of Progressive Hearing Impairment

There is a large and rapidly increasing amount of published data showing that cisplatin-related hearing loss is not limited to a loss of outer hair cells but instead also involves an early and ongoing loss of synapses between inner hair cells (IHCs) and spiral ganglion neurons (SGNs) [56]. Each IHC has 15–30 glutamatergic afferent terminals, each of which is associated with a unique presynaptic structure called ribbon synapses. These synapses allow for rapid and continuous release of the neurotransmitters necessary for auditory signal coding [57,58]. Nacher-Soler et al. found that the auditory synapse is the most susceptible cochlear structure due to cisplatin toxicity in a mouse model, with disappearance of ribbon synapses occurring prior to outer hair cell death and to elevation of auditory thresholds following exposure to low-dose cisplatin [59]. In a multisession mouse model of CIO, synaptic loss between IHCs and SGNs was identified as an early indicator of ototoxicity, with a significant latency delay in ABR wave I detectable after a single treatment cycle even before measurable threshold elevation [60]. This functional signature (reduced ABR wave I amplitude with preserved DPOAE) is consistent with the pattern of cochlear synaptopathy described in noise-exposed and aging cochleae, and may underlie the phenomenon of ‘hidden hearing loss’ in patients with apparently normal audiometric thresholds [61]. Mechanistically, cisplatin-induced cochlear synaptopathy appears to be mediated in part through nitrative stress. Treatment with cisplatin increases peroxynitrite levels in the cochlea, leading to nitration of synaptosomal proteins and disruption of ribbon synapse architecture [56]. Mass spectrometry-based proteomic analysis of cochlear synaptosomes from cisplatin-treated mice identified 102 proteins with reduced abundance and 249 with increased abundance after cisplatin exposure [56]. Critically, synapse loss is followed by the retraction of SGN nerve terminals, and over extended time periods, delayed the degeneration of spiral ganglion neurons, a process that may be driven by loss of trophic support from hair cells and can occur months to years after the initial synaptic injury [32,62]. This delayed neurodegeneration, driven by the sustained presence of platinum in cochlear tissues, is a plausible mechanism for the late progression of auditory impairment documented clinically.

## 7. Genetic and Individual Susceptibility Factors

Considerable interindividual variability in susceptibility to CIO exists beyond what can be explained by cumulative dose and age alone, strongly suggesting a genetic component to ototoxic risk [9,19]. Several pharmacogenetic polymorphisms have been identified to have associations with differential cisplatin-induced ototoxicity, including polymorphisms within the coding sequence of transport proteins for cisplatin. Specifically, polymorphisms in organic cation transporter 2 (OCT2, SLC22A2), the copper transporter 1 (CTR1, SLC31A1), and the multidrug resistance-associated protein 2 (MRP2, ABCC2), have been associated with altered platinum uptake into, or efflux from, cochlear cells [19,28]. CTR1 (SLC31A1) is especially noteworthy as it is a high-affinity copper import transporter that is highly expressed in the stria vascularis and has been identified as a major active transport pathway for the entry of cisplatin into cochlear cells. Variants that result in higher expression or activity of CTR1 could lead to higher levels of platinum accumulation in the cochlea and enhanced ototoxicity in patients. CTR1 has been shown to be a rational pharmacological target, as it can be targeted to reduce intracellular cisplatin accumulation and provide partial otoprotection without compromising the antitumor efficacy in some settings, as demonstrated in preclinical studies [28]. Additionally, polymorphisms within genes involved in the body’s response to oxidative stress (e.g., glutathione S-transferases [GSTP1, GSTM1, GSTT1] and thiopurine methyltransferase [TPMT]) have been consistently associated with a risk of ototoxicity in both pediatric and adult populations. In a landmark genome-wide association study of children with CIO, variants in ACYP2 were identified as a significant predictor of CIO in children [9]. The higher incidence and severity of CIO in children compared with adults may reflect not only the immature state of the blood–labyrinth barrier, but also pharmacokinetic differences in drug distribution, the greater proportion of cochlear platinum retained per unit of drug administered, and the longer lifespan over which cochlear damage can evolve and progress in pediatric patients [17,37]. As previously reported, the developmental stage of the auditory system and the intrinsically greater sensitivity of the immature cochlea must be considered when assessing ototoxic risk and planning audiological surveillance [7,15].

## 8. Clinical Implications: The Need for Long-Term Audiological Follow-Up

The convergence of evidence demonstrating indefinite cochlear platinum retention, post-treatment progression of auditory dysfunction, and the protracted timeline of delayed spiral ganglion neurodegeneration has direct and important implications for clinical practice. Patients who have undergone treatment with cisplatin benefit substantially from hearing evaluations conducted as part of their long-term follow-up with appropriate audiologic testing. This testing will provide invaluable information concerning the extent of any late-onset, significant cisplatin-induced hearing loss and allow for the early identification of any resultant cochlear damage, thereby providing the opportunity for proper aural rehabilitation. It has been established that at least 18 months after the last dose of cisplatin, cisplatin is still detectable in the cochlea [12,37,38,39]. It has also been reported that the systemic retention of cisplatin in the circulation of treated patients is over 20 years [35,42]. Thus, the scientific and clinical significance of the long-term follow-up of hearing conditions among patients who received cisplatin is justified [32]. Large proportions of new cases of ototoxic hearing loss as a result of the late manifestation of the ototoxicity of cisplatin are still being diagnosed in the long-term follow-up of cisplatin-treated patients [63,64]. Many international organizations (e.g., International Society of Pediatric Oncology (SIOP) and the Children Oncology Group (COG)) recommend the long-term audiometric follow-up of patients who received ototoxic effects of platinum-based drugs, and recommend that the patient group undergo regular ototoxicity audiometric monitoring throughout their lifespan, with a particular focus on the measurement of the extended high-frequency thresholds [32]. Audiometric monitoring needs to be performed at standard frequencies (0.25–8 kHz) but also at higher frequencies (up to 16–20 kHz) with calibrated high-frequency audiometry, which has been shown to be more sensitive in detecting CIO at an early stage [40]. Distortion product otoacoustic emissions should be added to determine the functionality of outer hair cells without reference to audiometric thresholds. In patients with normal pure-tone audiometry thresholds but difficulty understanding speech in noise (a common manifestation of cochlear synaptopathy), additional assessments of speech-in-noise perception and electrophysiological testing, including analysis of ABR wave I amplitude, may provide complementary diagnostic information [60,61].

## 9. Otoprotective Strategies

The mechanistic perspectives discussed in this paper provide several possible otoprotective intervention targets. Antioxidant approaches involving the NOX3/ROS axis have proven to be highly effective in animal models; systemic or local administration of compounds such as N-acetylcysteine, ebselen, sodium thiosulfate, and D-methionine has been investigated. Sodium thiosulfate, given intravenously 6 h after each cisplatin infusion, showed a significant reduction in the incidence of Brock grade ≥ 1 hearing loss from 63% to 33% (relative risk 0.52; *p* = 0.002) in the phase III SIOPEL 6 randomised trial (n = 109 children with standard-risk hepatoblastoma) without affect overall or event-free survival, although its systemic use in metastatic cancer is still under investigation due to concerns about potential interference with cisplatin’s antineoplastic efficacy depending on timing and dosing, although this is not universally observed [65]. This limitation could be overcome by intratympanic administration of otoprotective agents to limit the delivery of these agents to the cochlea. Blocking cisplatin uptake into cochlear cells through transporters, specifically OCT2, CTR1 or other uptake systems in the inner ear and not tumor cells, is a pharmacologically rational strategy and supported by preclinical studies [28,47,55]. However, selective inhibition in the cochlea without affecting tumor uptake remains experimental and has not yet been translated into clinical therapy. The stria vascularis, which is the main route of entry and retention of cochlear platinum, is a particularly promising therapeutic target: agents that inhibit the accumulation of cisplatin in the stria vascularis without affecting its systemic delivery to tumor cells would selectively decrease ototoxicity [34,66,67]. The recent studies involving the application of ICP-MS-based methods to screen candidate molecules on their capacity to inhibit cochlear platinum uptake in animal models can accelerate the process of identifying such compounds [37]. Given the evidence for progressive cochlear synaptopathy as a mechanism of long-term auditory deterioration, neuroprotective strategies aimed at preserving spiral ganglion neurons and promoting synapse maintenance, including the use of neurotrophins such as NT-3 and BDNF, merit investigation in the context of CIO, as these therapies are not yet clinically approved. Genetic screening prior to treatment to identify patients at high risk based on pharmacogenetic variants could allow for the individualization of cisplatin doses and otoprotective strategies, minimizing cochlear damage without compromising oncological outcomes [9,19]; however, this approach remains under research and is not yet part of standard clinical practice.

## 10. Conclusions

The mechanism of progressive hearing loss after cisplatin chemotherapy is a multifactorial process that is mediated by various interacting mechanisms. The prolonged retention of platinum in the cochlea, in contrast to the effective excretion of the drug in other organs, produces a long-lasting toxic exposure that continues even after discontinuation of treatment. Preferential accumulation of cisplatin in the stria vascularis partially impairs the endocochlear potential and compromises the function of surviving outer hair cells. Continued oxidative stress, inflammation, and genotoxic injury of retained platinum species contribute to the further death of cochlear cells in the months and years after treatment. Cochlear synaptopathy, such as early loss of ribbon synapses and late neurodegeneration of the spiral ganglion, has a significant role in the progressive loss of auditory function. Finally, personal genetic influences regulate cisplatin delivery and cellular sensitivity, which are part of the interindividual differences in ototoxic effects. All of these mechanisms support a paradigm shift in the audiological surveillance of cisplatin-treated patients: post-treatment monitoring in the short-term is not sufficient, and audiological monitoring throughout their lifetime is required to embrace the whole range and time-course of CIO. At the same time, mechanistic knowledge offers a logical foundation of otoprotective measures aimed at cisplatin uptake in the cochlea, oxidative stress, and neuronal survival. The key issue in the field is how to reconcile effective otoprotection with maintenance of antineoplastic effect, and further translational studies that combine pharmacokinetics, molecular biology, and clinical audiological science will be necessary to address this.

## Figures and Tables

**Table 1:** Risk factors for cisplatin-induced ototoxicity: Summary of evidence.

Risk Factor	Strength of Evidence	Key Data	Key References
TREATMENT-RELATED FACTORS			
Cumulative cisplatin dose	Strong	Incidence > 50% with cumulative dose > 400 mg/m^2^ (Bokemeyer et al. [20]); ~20% at standard testicular cancer doses. Bertolini et al. (n = 120): ototoxicity most often observed at ≥400 mg/m^2^ (median cumulative dose 400 mg/m^2^); 37% grade ≥ 2 in cisplatin-only group. Most consistent predictor across all age groups.	[8,12,20]
Cranial irradiation	Strong	Additive ototoxic effect via ROS generation. Cumulative dose of cisplatin + radiation independently associated with SNHL.	[15,18]
Administration schedule	Strong	Amount of cisplatin per single dose (not cumulative dose) was the strongest independent predictor of hearing loss in multivariate analysis (n = 153 pediatric patients; 72.6% incidence). Continuous infusion associated with markedly lower ototoxicity vs. bolus in retrospective series (1/21 patients receiving ≥ 400 mg/m^2^ developed significant ototoxicity). In a prospective clinical study of adults with head and neck squamous cell carcinoma receiving chemoradiation (Fernandez et al., 2025) [21], weekly low-dose cisplatin (<75 mg/m^2^) was associated with significantly lower hearing loss compared to the every-3-weeks high-dose schedule (≥75 mg/m^2^): CTCAE grade ≥ 2 hearing loss occurred in 18% vs. 50%, respectively, without difference in 2-year survival outcomes.	[21,22,23,24]
Concomitant ototoxic drugs	Moderate	Aminoglycosides and loop diuretics potentiate cochlear damage. Furosemide + cisplatin causes rapid, massive OHC loss in animal models.	[2,8]
Vincristine co-administration	Moderate	Vincristine exposure independently associated with increased hearing loss risk across multiple cohorts. Mechanism not fully established; possible disruption of cochlear microtubule dynamics or altered cisplatin pharmacokinetics. Evidence is growing but limited by co-occurrence with cisplatin and other agents.	[25,26]
PATIENT-RELATED FACTORS			
Younger age at treatment	Strong	Children < 5 years: OR = 21.17 (95% CI 2.48–180.94) vs. >15 years. Mean age at Brock grade 3: 4.5 years vs. 11.5 years at grade 1 (*p* = 0.02).	[8,13]
Renal dysfunction	Moderate	Impaired clearance increases systemic platinum AUC and prolongs cochlear exposure. Pretreatment GFR recommended as a baseline parameter.	[2,27,28]
Male sex	Moderate	Males at significantly greater risk (*p* = 0.005; OR = 4.812). May reflect sex differences in cochlear antioxidant defense. In females, potential otoprotective effect of estrogens.	[13]
Pre-existing hearing loss	Moderate	Pretreatment hearing level is an independent predictive factor for post-treatment hearing capability in head and neck cancer patients. Baseline audiometry recommended as standard of care before treatment initiation.	[29,30]
Ventriculoperitoneal (VP) shunt	Moderate	Significant hearing loss in pediatric medulloblastoma patients treated with cisplatin + craniospinal irradiation (n = 33; 100% hearing loss in shunted patients vs. 70% without shunt; *p* = 0.0008). Proposed mechanism: VP shunt-induced alterations in CSF pressure modify cochlear endolymphatic fluid dynamics via a patent cochlear aqueduct, potentiating cisplatin-related cochleotoxicity. Evidence currently limited to a single retrospective cohort; prospective confirmation required.	[31,32]
GENETIC/PHARMACOGENETIC FACTORS			
Genetic variants (ACYP2, GSTP1, TPMT, OCT2, LRP2)	Moderate	The effect of a genetic variant may be more apparent when it occurs alongside other polymorphisms or specific clinical factors.	[9,33]
OTHER FACTORS			
Cardiovascular risk factors	Emerging	Long-term elevated circulating platinum has been associated with cardiovascular late effects (including increased risk of hypertension and dyslipidemia) in testicular cancer survivors (Boer et al., n = 1289); it is important to note that this association is specific to cardiovascular outcomes and does not directly demonstrate a causal relationship with hearing decline. Metabolic comorbidities may compound cochlear aging and accelerate audiological decline over time; no direct OR for hearing loss confirmed in peer-reviewed literature to date.	[34,35]

Evidence strength classifications (Strong/Moderate/Emerging) represent a study-specific framework developed by the authors for the purpose of this review and are not derived from a formal validated grading system (e.g., GRADE). Strong = confirmed in multiple independent cohorts and/or meta-analyses; Moderate = replicated in at least two independent studies; Emerging = preliminary or single-cohort data requiring confirmation. AUC = area under the curve; GFR = glomerular filtration rate; OHC = outer hair cells; OR = odds ratio; SNHL = sensorineural hearing loss.

## Data Availability

No new data were created or analyzed in this study.

## Abbreviations

The following abbreviations are used in this manuscript:

4-HNE	4-Hydroxynonenal
ABR	Auditory brainstem response
ACYP2	Acylphosphatase 2
ASHA	American Speech-Language-Hearing Association
AUC	Area under the curve
BDNF	Brain-derived neurotrophic factor
BLB	Blood–labyrinth barrier
BODIPY FL	Boron-dipyrromethene fluorescent (cisplatin analogue)
CIO	Cisplatin-induced ototoxicity
COG	Children’s Oncology Group
CSF	Cerebrospinal fluid
CTR1 (SLC31A1)	Copper transporter 1
DPOAE	Distortion product otoacoustic emission
EP	Endocochlear potential
ER	Endoplasmic reticulum
FDA	Food and Drug Administration
GFR	Glomerular filtration rate
GSH	Glutathione
GSTM1	Glutathione S-transferase Mu 1
GSTP1	Glutathione S-transferase Pi 1
GSTT1	Glutathione S-transferase Theta 1
ICP-MS	Inductively coupled plasma-mass spectrometry
IHC	Inner hair cell
MRP2 (ABCC2)	Multidrug resistance-associated protein 2
NOX3	NADPH oxidase isoform 3
NRF2	Nuclear factor erythroid 2-related factor 2
NT-3	Neurotrophin-3
OC	Organ of Corti
OCT2 (SLC22A2)	Organic cation transporter 2
OHC	Outer hair cell
PUFAs	Polyunsaturated fatty acids
ROS	Reactive oxygen species
SGN	Spiral ganglion neuron
siRNA	Short interfering RNA
SIOP	International Society of Pediatric Oncology
SNHL	Sensorineural Hearing Loss
STAT1	Signal transducer and activator of transcription 1
SV	Stria vascularis
TPMT	Thiopurine methyltransferase
TRPV1	Transient receptor potential vanilloid 1
VP shunt	Ventriculoperitoneal shunt
